# The Discrete Multi-Hybrid System for the Simulation of Solid-Liquid Flows

**DOI:** 10.1371/journal.pone.0124678

**Published:** 2015-05-11

**Authors:** Alessio Alexiadis

**Affiliations:** School of Chemical Engineering, University of Birmingham, Birmingham, United Kingdom; University of Washington, UNITED STATES

## Abstract

This study proposes a model based on the combination of Smoothed Particle Hydrodynamics, Coarse Grained Molecular Dynamics and the Discrete Element Method for the simulation of dispersed solid-liquid flows. The model can deal with a large variety of particle types (non-spherical, elastic, breakable, melting, solidifying, swelling), flow conditions (confined, free-surface, microscopic), and scales (from microns to meters). Various examples, ranging from biological fluids to lava flows, are simulated and discussed. In all cases, the model captures the most important features of the flow.

## Introduction

**Fig 1:**
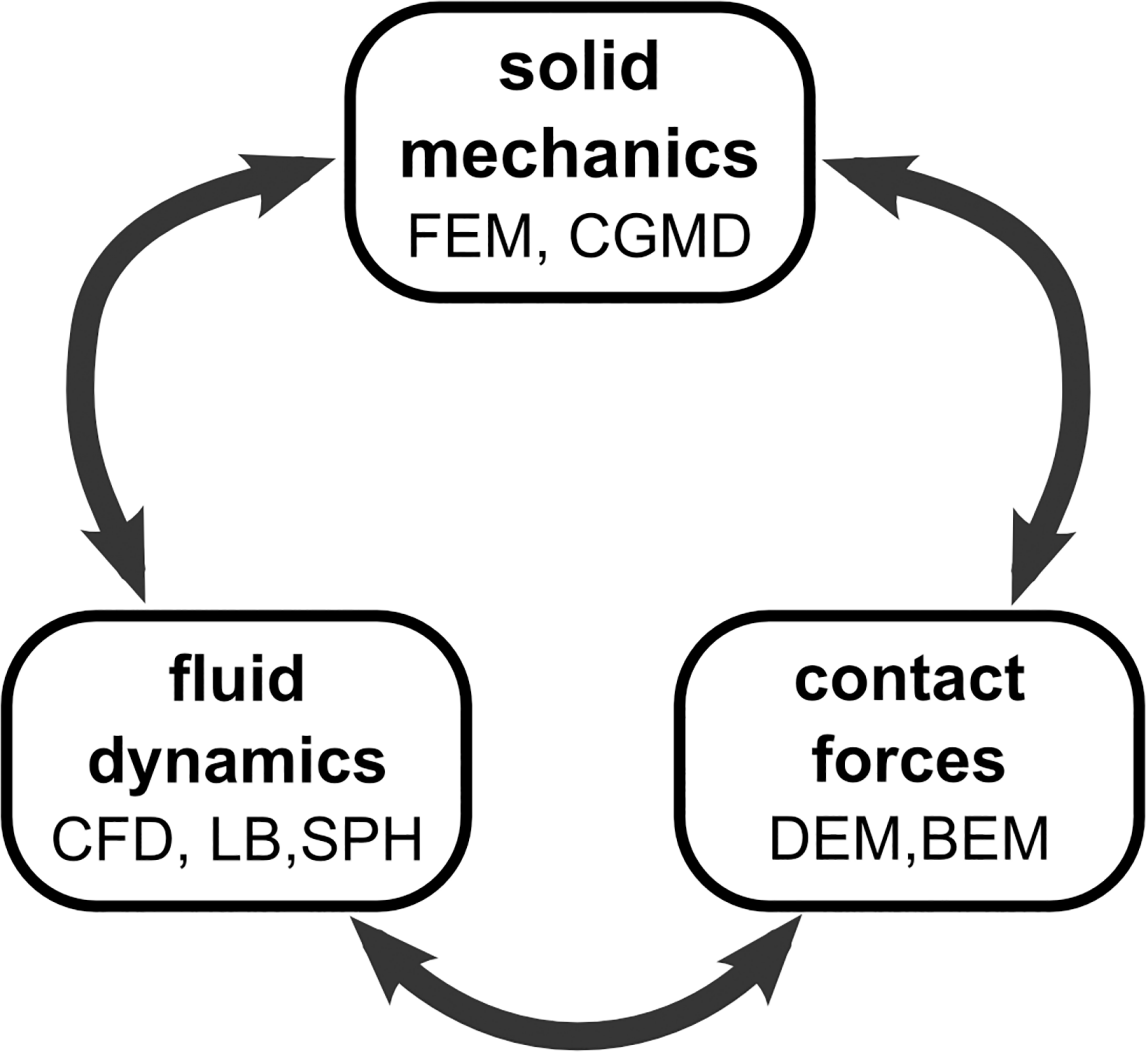
Fluid dynamics, contact forces and solid mechanics in solid-liquid flows and some modelling techniques available for each case.

The flow of solid-liquid suspensions is a generic problem which poses many challenges to scientists and industrialists across many different areas. Applications range widely from processing of food and pharmaceuticals, through oil and mining industries, to blood and biological applications. Such flows involve a large array of complex phenomena on a wide range of scales, and the reciprocal interaction of liquid and dispersed solids creates a very complex dynamics, which often includes particle deformation, breakage, degradation, melting, swelling, erosion, aggregation etc. The variety of phenomena occurring in these flows can be divided in three main categories mutually linked in a feedback mechanism (see Fig 1): fluid phenomena, solid phenomena and contact phenomena. Traditionally, specific modelling techniques have been developed by focusing on certain specific aspects of the flow and simplifying the others. Computational Fluid Dynamics (CFD), for instance, accurately describes the fluid dynamics, but the solids phase is simplified by the point-particle assumption. Other techniques, such as the Discrete Element Method (DEM) provide a good account of the inter-particle contact forces, but it cannot handle phenomena such as solid-liquid mass transfer or melting/solidification (softening and melting of solid materials has been modelled with DEM [1], but the dynamics of the liquid, once melting has occurred, requires a different modelling technique). Computational methods dedicated to solid mechanics, on the other hand, describes the elastic and plastic deformations in the solid, but the external stresses coming from the fluid must be known in advance and provided as boundary conditions.

In order to achieve a more sophisticated description of these systems, hybrid models have been suggested. There are, however, some major issues that have, so far, limited the use of this type of modelling in engineering. The variety of models available for each phenomenon and the possibility of combining them in a hybrid approach, for instance, have led to an uncontrolled proliferation of hybrid models. There are studies, just to name a few, where DEM is coupled with the CFD [2]; where Lattice Boltzmann (LB) is coupled with DEM [3], Smoothed Particle Hydrodynamics (SPH) with Molecular Dynamics (MD) [4], DEM with SPH [5] and MD with CFD [6]. Each of these has certainly its advantage, but the variety of approaches has created a very heterogeneous and disconnected environment, which, eventually, represents a barrier to the diffusion of these methodologies outside the academic world and, sometimes, even outside the narrow circle of specialists of a certain specific method.

**Fig 2:**
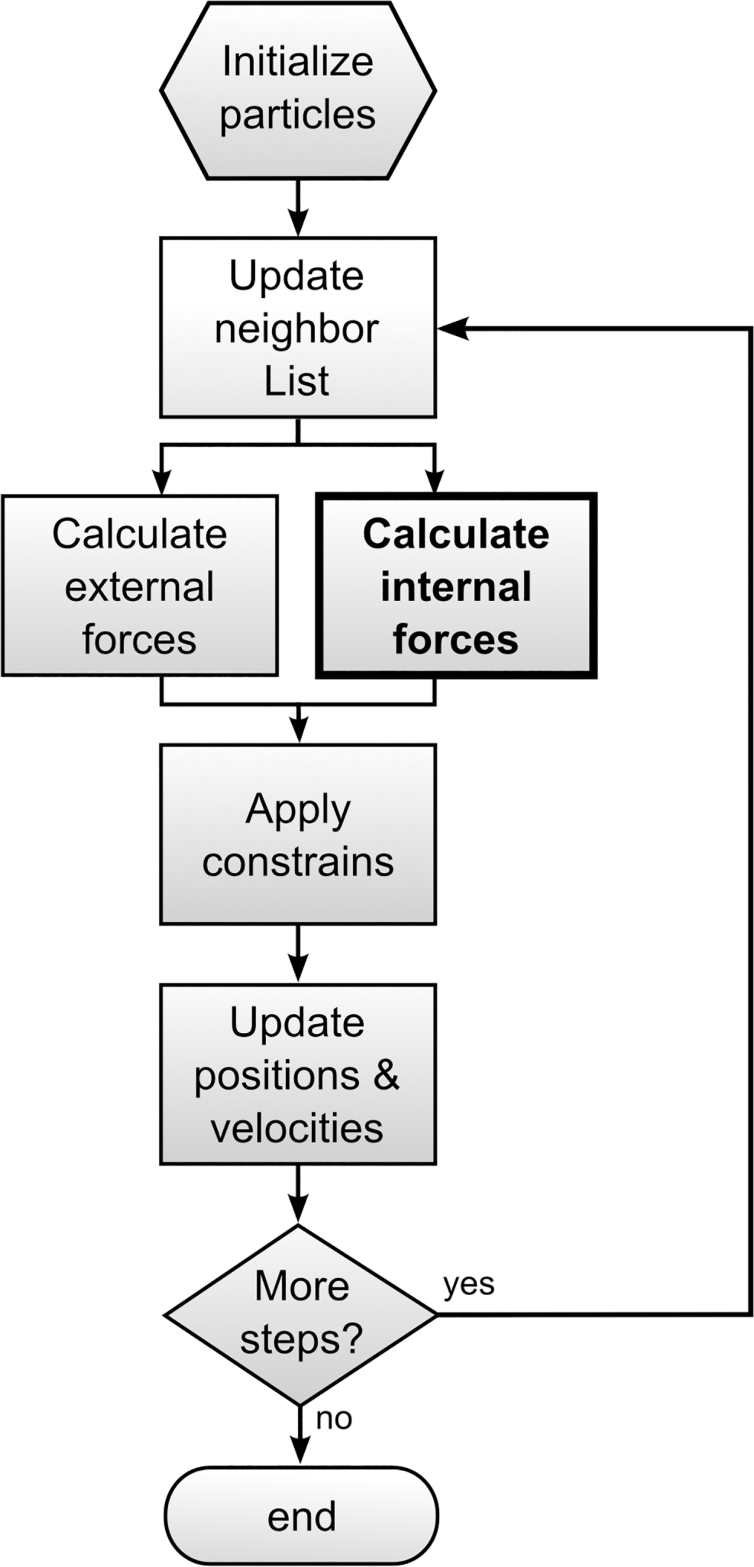
Structure of a typical particle-based algorithm with the internal forces routine highlighted.

The goal of this paper is not so much to propose a new hybrid model by coupling two methods that have so far escaped the “hybrid-frenzy”, but rather to create a common framework that supports and facilitates the linkage of different models in a hybrid fashion. The objective, ultimately, is to *model-by-models*, that is the ability to link, as if they were Lego bricks, the most suitable modelling techniques in order to achieve a complete representation of the system under investigation. Models such as CGMD, SPH and DEM share a common discrete or *particle-based* paradigm and, for this reason, seem well suited as basis for a unified modelling framework. If we observe, in fact, the typical flowchart of a CGMD, SPH or DEM code (Fig 2), the only difference is the routine that explicitly calculates the internal forces. In SPH these are hydrodynamic forces, in DEM contact forces, and in CGMD deformation forces, but, except for that, the algorithm is practically the same in all cases.

A systematic literature review on SPH, CGMD, DEM and hybrid methods is beyond the scope of this paper. The interested reader can refer to [7–11] for a more comprehensive survey.

Before concluding this section, a few words on the terminology are necessary. In this study, we deal with two types of discrete entities both defined as “particles”: *real* particles, which are minute portions of solid matter dispersed in the flow, and *computational* particles, which are notional particles used to discretize both the fluid and the real particles. A real particle, therefore, is made of several computational particles. In order to avoid confusion the adjectives *real* or *dispersed* are used to indicate the former, and *computational* or *elemental* to indicate the latter. Additionally, the terms SPH-particle, DEM-particle or CGMD-particle are employed to indicate elemental particles used by a specific model.

## Towards a Unified Computational Framework

In particle-based modelling, the information is lumped into discrete entities that can be seen in two alternative ways: as Lagrangian nodes of a moving mesh whose position is updated during the simulation according to certain rules, or as “lumps” of matter that move according to the laws of classical mechanics

$$m_{i}\frac{\text{d}v_{i}}{\text{d}t}=m_{i}\frac{d^{2}r_{i}}{dt^{2}}=\sum_{i\neq j}F_{i,j}+\sum F_{E},$$ (1)

where *m* is the mass of particle *i*, **v** its velocity, **r** its position, **F**_*E*_ the external forces, and **F**_*i*,*j*_ the internal or inter-particle forces (see Fig 2). By coupling techniques such as CGMD, SPH and DEM, these two points of view coincide and we can talk of *node-particle duality*. Our notional particles are Lagrangian nodes and, at the same time, Eq (1) is used to update their position at each time step. Eq (1), therefore, represents the mathematical foundation of the unification method and the differences among CGMD, SPH and DEM are condensed in the explicit **F**_*i*,*j*_ term of Eq (1). The expression of this term in the three models is discussed respectively in the next three Sections. The Section “Linking the three models” explains how the models are linked together in our unified framework.

### Smoothed Particle Hydrodynamics

The fundamental idea behind the SPH method lies in the mathematical identity

$$f\left(r\right)=∭f\left(r^{'}\right)\delta \left(r-r^{'}\right)dr^{'},$$ (2)

where *f*(**r**) is a generic function defined over the volume V, the vector **r** is a three-dimensional point in V and *δ*(**r**) is the three-dimensional delta function. In the SPH formalism, the delta function is approximated by a function *W* called the smoothing kernel with a characteristic width *h* (smoothing length) such that

$$\underset{h\to 0}{\text{lim}}W(r,h)=\delta \left(r\right),$$ (3)

which brings to the approximation

$$f\left(r\right)\approx ∭f\left(r^{'}\right)W\left(r-r^{'},h\right)dr^{'}.$$ (4)

Eq (4) can be discretised over a series of particles of mass *m* = *ρ*(**r**)**dr** obtaining

$$f\left(r\right)\approx \sum_{i}\frac{m_{i}}{\rho_{i}}f\left(r_{i}\right)W\;\left(r-r_{i},h\right),$$ (5)

where *f*(**r**_**i**_), *m*_*i*_ and *ρ*_*i*_ are the mass and density of the *i* ^th^ particle, and *i* ranges over all particles within the smoothing kernel. Eq 5 represents the discrete approximation of a generic continuous field and can be used to approximate the Navier-Stokes equation at a set of Lagrangian points, which can be thought as particles characterized by their own mass, velocity, pressure and density

$$m_{i}\frac{dv_{i}}{dt}=\sum_{j}m_{i}m_{j}\left(\frac{P_{i}}{\rho_{i}^{2}}+\frac{P_{j}}{\rho_{j}^{2}}+\Pi_{i,j}\right)\nabla_{j}W_{i,j}+\sum F_{E},$$ (6)

where *W*_*i*,*j*_ means *W*(**r**_**j**_ **-r**_**i**_, *h*), ∇_*j*_ denotes the gradient of the kernel with respect of the coordinate *r*_*j*_, *P* is the pressure, and ∏_*i*,*j*_ introduces the viscosity forces. Various expressions for the tensor ∏_*i*,*j*_ are available in the literature. In our calculation, we use both Monaghan’s (Re > 1) [12] and Morris’ [13] (Re < 1) formulations. By comparing Eq (1) and Eq (6), we can see the form of the **F**_*i*,*j*_ term in the case of SPH. At each time step, Eq (6) is used to update the velocities of the fluid particle, while their density can be calculated either by Eq (5), considering *ρ* as a normal scalar field, or, as done in this work, by means of the SPH approximation of the continuity equation

$$\frac{d\rho_{i}}{dt}=\sum_{j}m_{j}v_{i,j}\nabla_{j}W_{i,j},$$ (7)

where **v**_*i*,*j*_ = **v**_*i*_ **-v**_*j*_.

In its original version, the SPH method was derived for compressible flows. Incompressible, and computationally more expensive, versions have been subsequently proposed, but for problems at low Mach number, the weakly-compressible approach brings only small density variations and can be safely used. Eq (6) requires an equation of state that relates *ρ* and *P*; in this paper, we use Tait’s equation of state, which has been specifically devised to model water

$$P(\rho )=\frac{c_{0}\rho_{0}}{7}\left[{\left(\frac{\rho }{\rho_{0}}\right)}^{7}-1\right],$$ (8)

where *c*_0_ and *ρ*_0_ are, respectively the sound speed and density at zero applied stress. As mentioned, this section only gives a brief introduction of SPH for fluids, more information can be found in [4].

### Coarse-Grained molecular dynamics

This section provides a brief introduction of some aspects of MD and CGMD relevant to the method proposed here, refer to [14] for more details. Molecular dynamics is a form of investigation where the motion and the interaction of a certain number of computational atoms or molecules are studied. In classical MD simulations atoms move according to the Newtonian equations of motion

$$m_{i}\frac{d^{2}r_{i}}{dt^{2}}=-\frac{\partial }{\partial r}U_{tot}(r_{1},r_{2},\ldots r_{N})+\sum F_{E},$$ (9)

where *U*_*tot*_ is the total interatomic potential, whose negative gradient provides the **F**_*i*,*j*_ forces in Eq (1). The interatomic potential can be divided into two main parts: non bonded and intramolecular. Non bonded forces are usually represented by the so-called Lennard-Jones potential, while the intermolecular forces are often divided in subgroups

$$U_{intermolecular}=U_{bond}+U_{angle}+U_{dihedral}.$$ (10)

Each of these potentials can have different forms. For simplicity, in this study, we only consider harmonic potentials, but more complicated expressions such as FENE, Morse or quartic can be easily introduced. The harmonic potentials used for the calculations in this work are:

$$U_{bond}=k_{b}{\left(r-r_{0}\right)}^{2},$$ (11)

where *k*_*b*_ is the Hookean coefficient and *r*_0_ the equilibrium distance,

$$U_{angle}=k_{a}{\left(\theta -\theta_{0}\right)}^{2},$$ (12)

where *k*_*a*_ is the angular Hookean coefficient and *θ*_0_ the equilibrium angle,

$$U_{dihedral}=k_{d}{\left(\phi -\phi_{0}\right)}^{2},$$ (13)

where *k*_*d*_ is the torsional Hookean coefficient and *ϕ*_*0*_ the equilibrium dihedral angle (discussed below).

**Fig 3:**
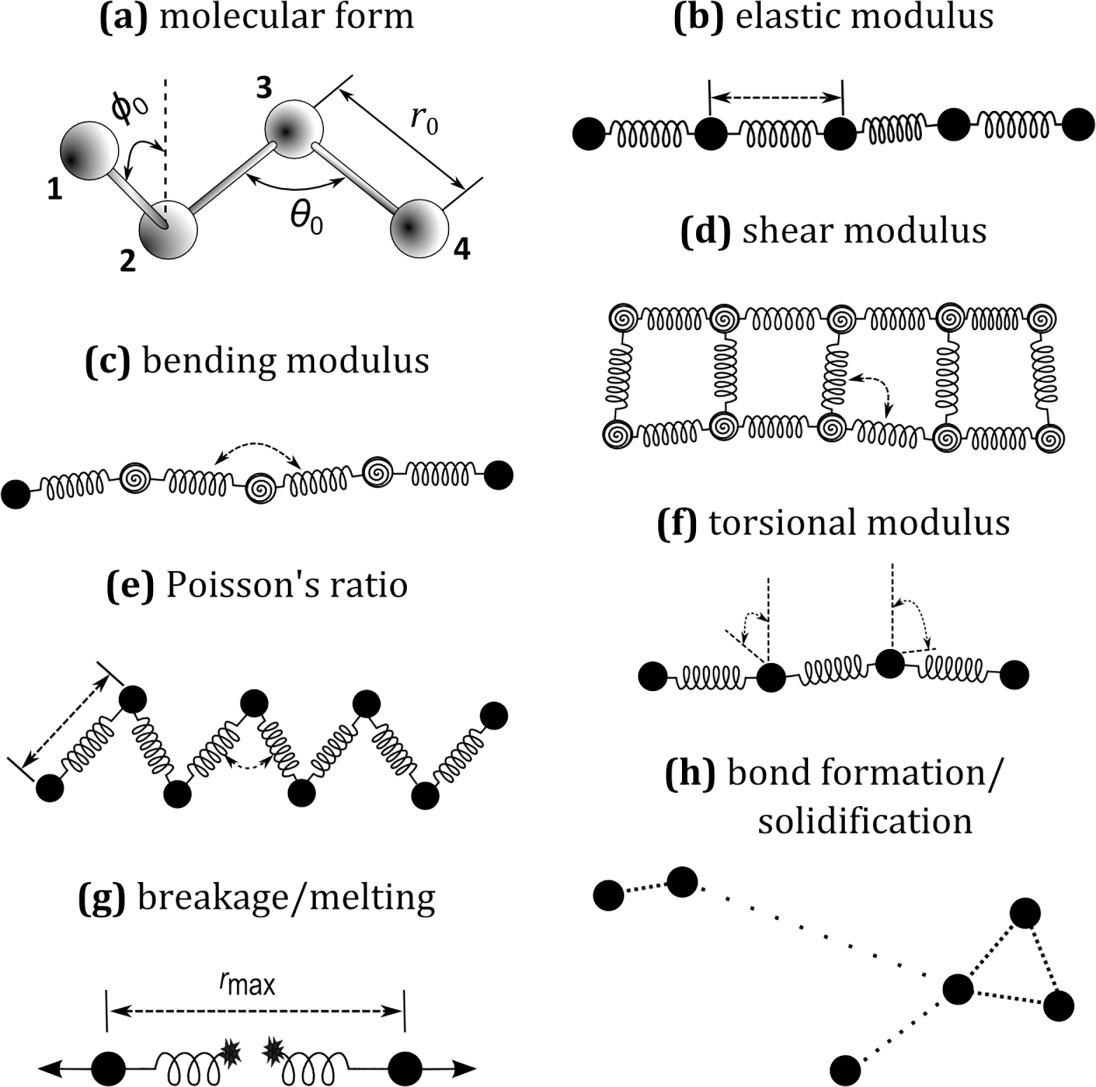
Coarse-grain modelling of solids and its molecular origin.

Eqs (11), (12) and (13) are the basis for the ball-and-stick representation of molecules that can be coarse-grained to model macroscopic solids (see Fig 3). It is important to highlight that here course-graining is brought to its extreme consequences. Normally, the term CGMD is used to indicate simulations that are coarse-grained, but still at the molecular scale. In this study, we apply a nominally smilar approach, but to considerably larger lumps of matter. This entails a number of differences expecially in relation to the notion of temperature. At the molecular level, in fact, temperature is defined as the second moment of the velocity distribution. The examples discussed in Section “Examples of applications and discussion” have a physical scale where this ‘brownian’ component is too small to be percieved as independent motion. This means that if we are interested in the temperature, we need to introduce heat as a separate macroscopic variable that obeys its own conservation equation as in Section “Solidification and melting” and “Lava flows”. In theory, we should, more correctly, have used the expression Coarse-graining Modelling instead of CGMD, but we prefer the latter because emphatizes the fact that the potentials are formally similar to those used in MD. Moreover, the fact that brownian motion has not be included in Eq (1) only depends on the choice of examples considered in this study. As explained in more detail in the conclusions, our methodology has the potential to cover a large variety of scales including those where CGMD mantains its molecular origin.

Fig 3A illustrates the molecular foundation of Eqs (11), (12) and (14). Atoms belonging to a certain molecule are bound together by means of forces, which tend to maintain two atoms at a certain specific distance *r*_*0*_ (see Eq 11), three atoms at a certain specific angle *θ*_*0*_ (see Eq 12) and four atoms at a certain specific dihedral angle *ϕ*_*0*_ (see Eq 13). The dihedral, or torsional, angle is the angle between the two planes generated by atoms 1-2-3 and atoms 2-3-4 in Fig 3A. As already mentioned this approach can be coarse-grained and employed to model different phenomena occurring in solids like stretching, bending or torsion of particles under the effect of external forces. The elastic modulus (Fig 3B) is connected to Eq 11 by considering coarse-grained portions (pseudo-particles) of the solid instead of atoms [15]. The bending modulus can be achieved by considering Eq 12 acting on a sort of ‘hinge’ as illustrated in Fig 3C. In order to simulate the effect of shear, the solid can be structured as indicated in Fig 3D and Eq 12 applied to the internal angles. A similar approach can be employed for the Poisson’s Ratio by arranging two layers of particles as in Fig 3E. The dihedral angle (Eq 13), on the other hand, can be used to simulate torsion as indicated in Fig 3F. Breakage and melting can also be included by assuming that, for instance, if the distance between two particles exceeds a certain maximum value *r*_max_ or the temperature (intended in macroscopic sense) exceeds a certain value, the bond is broken and the two particles separated (Fig 3G). Finally, solidification can be modelled by considering the formation of inter-particle bonds according to certain rules (Fig 3H).

Fig 3 illustrates the strategies that can be used to simulate various macroscopic solids with coarse-grained potentials formally similar to those used in MD. These strategies, moreover, can be combined together in order to cover the whole array of macroscopic phenomena occurring in solids. We can use, for instance, Eq 11 for simulate the elastic modulus and two sets of angular potentials (Eq 12), one for the bending modulus (Fig 1C) and another for the shear modulus (Fig 1D).

### Discrete Elements Method

**Fig 4:**
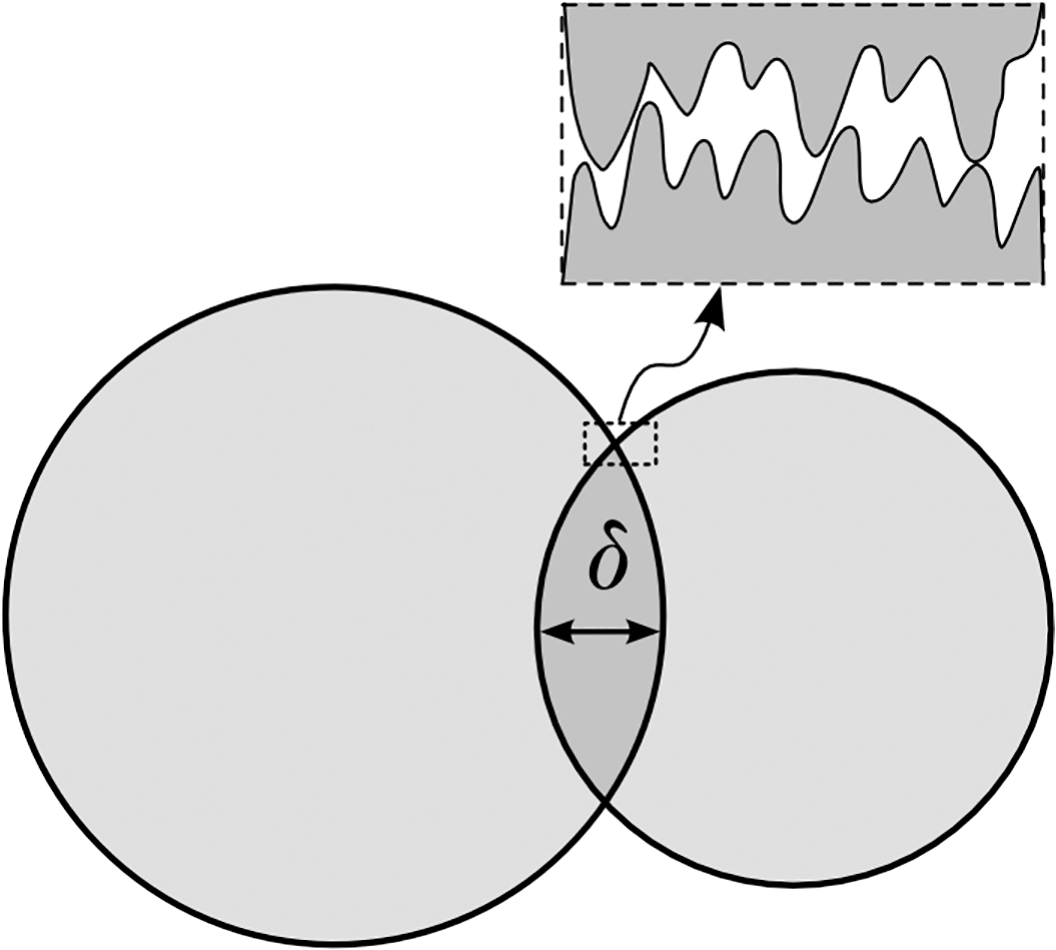
Contact between two DEM particles.

The dispersed phase is constituted of “grains”, which are put in motion by the fluid and interact by colliding with each other. The modelling of the contact forces generated by these collisions is the basis of DEM. It is helpful to highlight that DEM also represents a type of coarse-grained method with the difference that CGMD deals with the internal stresses occurring within a single grain, while DEM deals with the forces occurring at the contact point of two colliding grains. If it were possible to run a full MD simulation of two real colliding particles, we could fully characterize the interlocking of the surface asperities (see Fig 4) at the molecular level. Since this is not possible, DEM relates the contact forces to the overlap δ of two particles (Fig 4). As such, this overlap is a numerical artefact due to the time discretization of the equations of motion, but, despite this, it provides a good estimate of the real particle deformation. This section provides a brief introduction to DEM, see [16] for more details.

As already mentioned, Eq (1) is valid for SPH, CGMD and DEM with the only difference that the term **F**_*i*,*j*_ changes in each case. For DEM, however, the Newton equation of motion should be integrated also for the rotational degrees of freedom due to the possibility that tangential forces induce torsion or rotation in the grains. In our case, however, each grain is approximated by several elemental particles, which are bond together and cannot rotate independently. The resulting torque, therefore, depends exclusively on the forces acting on each computational particle and an independent equation for the balance of moment is not necessary.

The **F**_*i*,*j*_ forces in the case of DEM are contact forces of two types: normal and tangential forces. The simplest normal contact force model, which takes into account excluded volume and dissipation, involves a linear repulsive and dissipative force

$$f^{n}=k_{n}\delta +m_{eff}\;\gamma_{n}v_{n},$$ (14)

where *k*_*n*_ is a stiffness constant, γ_*n*_ a dumping coefficient, *v*_*n*_ the relative velocity in the normal direction and *m*_*eff*_ = *m*_*i*_ *m*_*j*_/(*m*_*i*_+*m*_*j*_) the effective mass of 2 colliding particles with mass *m*_*i*_ and *m*_*j*_. The results presented in Section 3 are based on Eq (14), but, conceptually, more complicated models involving nonlinear hysteretic forces, which take into account the possibility that at the contact point plastic deformation may take place, can be easily introduced.

Tangential forces are coupled to the normal forces trough Coulomb’s law

$$f^{t}\leq f_{C}^{s}=\mu^{s}f^{n},$$ (15)

where for the dynamic case one has dynamic friction with

$$f^{t}=f_{C}^{d}=\mu^{d}f^{n},$$ (16)

where μ^s^ is the static and μ^d^ the dynamic friction coefficient and, in general, μ^s^ *<*μ^d^. Below the Coulomb limit ($f^{t}\leq f_{C}^{s}$), one has static friction and the tangential force can be calculated with

$$f^{t}=-k_{t}\xi -m_{eff}\gamma_{t}v_{t},$$ (17)

where *k*_*t*_, γ_*t*_ and *v*_*t*_ are, respectively, the tangential stiffness, dumping coefficient and relative velocity, and *ξ* is the tangential displacement between two particles for the duration of the contact. Above the Coulomb limit, sliding friction becomes active and Eq (16) is used. In this study, we consider only simple linear tangential and normal forces. More complex formulations derived from Hertz or Mindlin-Deresiewicz theory can be introduced if necessary. The same can be said for other complex phenomena such as lubrication forces that can be included by choosing specific DEM potentials.

### Linking the three models

**Fig 5:**
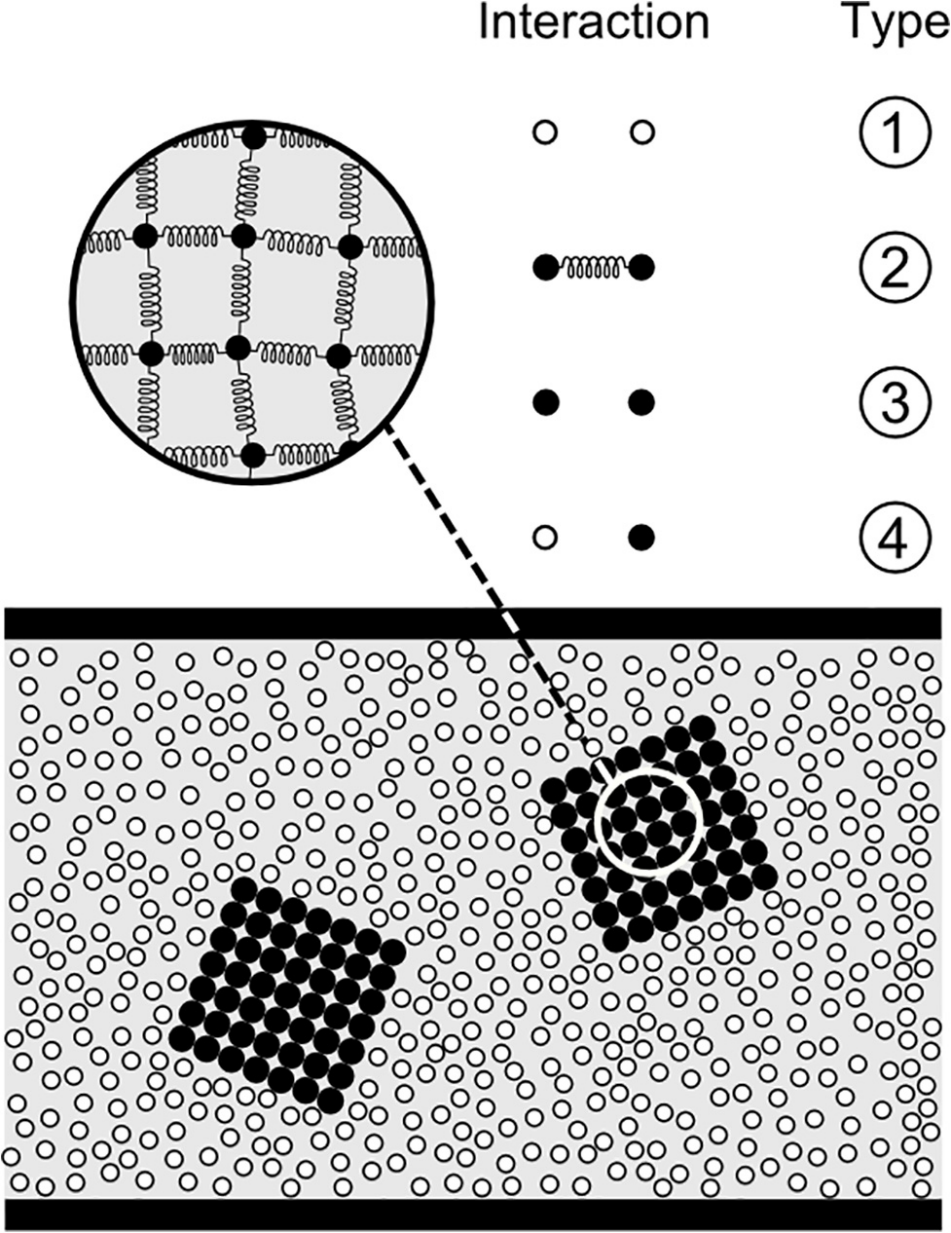
Types of interactions occurring between elemental particles.

The SPH, CGMD and DEM are based on a common particle paradigm and, therefore, the interactions among the liquid (SPH), the internal structure of the solid (CGMD) and the interface (DEM) is completely regulated by the **F**_*i*,*j*_ term in Eq (1). All the elemental particles coexist in the same domain and the linking among the three models can be simply achieved as the sum of all the forces involved. Fig 5 illustrates this point. In the picture, we have two solid cubes (real particles) constituted of 49 elemental particles (black circles) dispersed in a SPH fluid (white circles). There are, therefore, four types of interactions. The first (Type 1, in Fig 5) concerns the liquid-liquid SPH interactions and refers to the viscous and pressure forces in Eq (6). The second (Type 2) concerns the solid-solid CGMD interactions among elemental particles belonging to the same cube (Eqs 11–13). The third (Type 3, in Fig 5) concerns the solid-solid DEM contact interactions among elemental particles belonging to two different cubes (Eqs 14–17) and activated by inter-particle collision. There is, however, a forth type of interaction that occurs at the solid-liquid interface and it is not included in any of the previous items. In continuum modelling, this would be implemented by boundary conditions at the interface. In our discrete framework, however, also this type of interaction must be resolved in terms of forces **F**_*i*,*j*_. There are three main types of phenomena occurring at the solid-liquid interface [17, 18]: no-penetration, no-slip and continuity of stresses. In continuum mechanics, these conditions are often represented as

$$\left(\frac{\partial }{\partial t}u-v\right)\cdot n=0\;\left(\text{no}-\text{penetration}\right),$$ (18)

$$\left(\frac{\partial }{\partial t}u-v\right)\times n=0\;\left(\text{no}-\text{slip}\right)$$ (19)

and

$$\sigma_{s}n=\sigma_{f}\left(-n\right)\;\left(\text{continuity of stresses}\right)$$ (20)

where **n** is the normal to the boundary, **u** the displacement of the solid, **v** the velocity of the liquid, *σ*_*s*_ the stresses in the solid and σ_*f*_ in the fluid.

These conditions need to be ‘translated’ in terms of forces **F**_*i*,*j*_ in order to be introduced in our discrete framework. Here we use the same approach employed in SPH simulations at solid boundaries. The no-penetration conditions can be implemented by means of a repulsive Lennard-Jones potential between SPH and CGMD particles

$$f\left(r\right)=4\varepsilon \left[{\left(\frac{\sigma }{r}\right)}^{12}-{\left(\frac{\sigma }{r}\right)}^{6}\right],$$ (21)

where *r* is the distance between the particles, ε the depth of the potential well and σ the distance at which the inter-particle potential is zero. Eq 21 is truncated at a cut-off distance *r*_*c*_ = σ so that only repulsive forces are considered. In traditional modelling, the no-slip conditions are simply enforced by imposing zero relative velocity at the solid-liquid boundary. Here this result is achieved by superimposing fluid ghost particles above the solid particles at the interface [4]. Once both the no-penetration and no-slip boundary condition are enforced, the continuity of stress is automatically satisfied by Eq (1).

## Relation to Other Hybrids and Unification Theories

As discussed in Sections 4 and 5, the capabilities of our SPH-CGMD-DEM model go beyond the sum of its constituting parts. In the past, however, other hybrid models linking together two of the aforementioned techniques have been investigated. These models have their specific advantages, and objectives that are different from those of this study. It is important, therefore, to highlight the difference between our framework and these previous studies. The scope of this section is not to cover the whole literature in the field of hybrid modelling (this, alone, would require a book rather than a paper), but to focus only on a few selected methods that bring some resemblance with the technique proposed here.

### Previous SPH-MD hybrids

Hybrid models combining together SPH and Molecular Dynamics (MD) have been proposed in the past [4], but based on a completely different idea. The domain is divided in two separate regions, one for MD and the other for SPH. Each part represents a completely different time and length scale and the interaction between the two models is mediated by a common overlapping region that ensures consistency of momentum, energy and mass. In the present methodology, both models coexist in the same domain and at the same time. All the particles are free to move in the entire domain and to interact directly. As discussed in the conclusions, when phenomena at different scale are taken into account, this is not reflected in the physical position of the elemental particles, but rather in their interaction forces.

### Previous SPH-DEM hybrids

Hybrids combining SPH and DEM have also been investigated in the past [5, 19]. The DEM particles, however, were constituted of single elemental particles and, therefore, the effect of complex shapes and/or particle deformation/breakage was not considered. In principle, it would be possible to extend this approach to the case of complex shapes and brittle materials. For complex shapes, for instance, we could couple SPH with DEM clumps or aggregates [20] and the same idea could be extended to include breakage. The coupled deformation of layers of brittle and ductile solid materials (without liquid however), for instance, has already been investigated in this way [21].

The inclusion of a CGMD part in our model, however, allows the model to extend its capabilities in at least two new directions. Firstly, the modelling of the internal solid structure can be much more sophisticated. A very large variety of MD potentials, in fact, have been developed over the years and their functional form can be used to simulate bulk properties of a large variety of solid materials such as metals, polymers, proteins, salts etc. Additionally, the possibility to introduce bond breaking and bond formation allows the simulation of phenomena such as solidification and melting, which are beyond DEM capabilities. The second advantage of including CGMD is related to its multi-scale nature. As explained in the previous Section dedicated to CGMD, the coarse-graining is here brought to its extreme consequences in order to extend its use to macroscopic system. This means that, most of the time, we ignore the Brownian forces acting on elemental particles because we do not ‘see’ them at the scale under consideration. If we reduce the scale to the molecular level, however, Brownian motion needs to be considered. This can be easily introduced in our framework by including (see Fig 2) **F**_*E*_ Brownian random forces as, for instance, in Dissipative Particle Dynamics (DPD). The unified discrete framework proposed in this paper, therefore, provides a common basis not only for SPH, CGMD and DEM, but for any other discrete method with a structure similar to Fig 2, and, for this reason, we decided to name it the *discrete multi-hybrid system* (DMHS)

### The SPH-SPH unification theory

The SPH method can be used for both solids and liquids. For this reason, a unified framework entirely based on SPH has been proposed [22]. This idea does not come from traditional physics disciplines but from the field of computer animation. SPH, in fact, is today also used for computer animations in movies with special effects and in computer games. Computer scientists have developed, in the last years, many interesting ideas in this field; usually, however, their goal is not to predict actual phenomena, but to trick the audience into believing that the physical behaviour they see is plausible. In principle, it is not impossible to reformulate this approach within a physically more accurate representation of reality, but SPH is not the best choice for modelling phenomena involving particle collision. Contact and friction forces (Type 3 in Fig 5), in fact, are still an open question in SPH, while they are easily introduced by the inclusion of DEM in our multi-hybrid system.

## Examples of Applications and Discussion

Various examples are presented and discussed in this section. The goal is to show the flexibility of the DMHS in a variety of scenarios. For simplicity, all the calculations are in 2D and based on a relatively small number of particles. The examples in this Section must be considered a proof of concept about the type of problems that the DMHS can handle rather that a systematic study. In all the cases considered, however, we check that the results are consistent with the expected physics of the problem and that all significant phenomena are captured by the model.

### Cells, vesicles and capsules under various flow conditions

**Fig 6:**
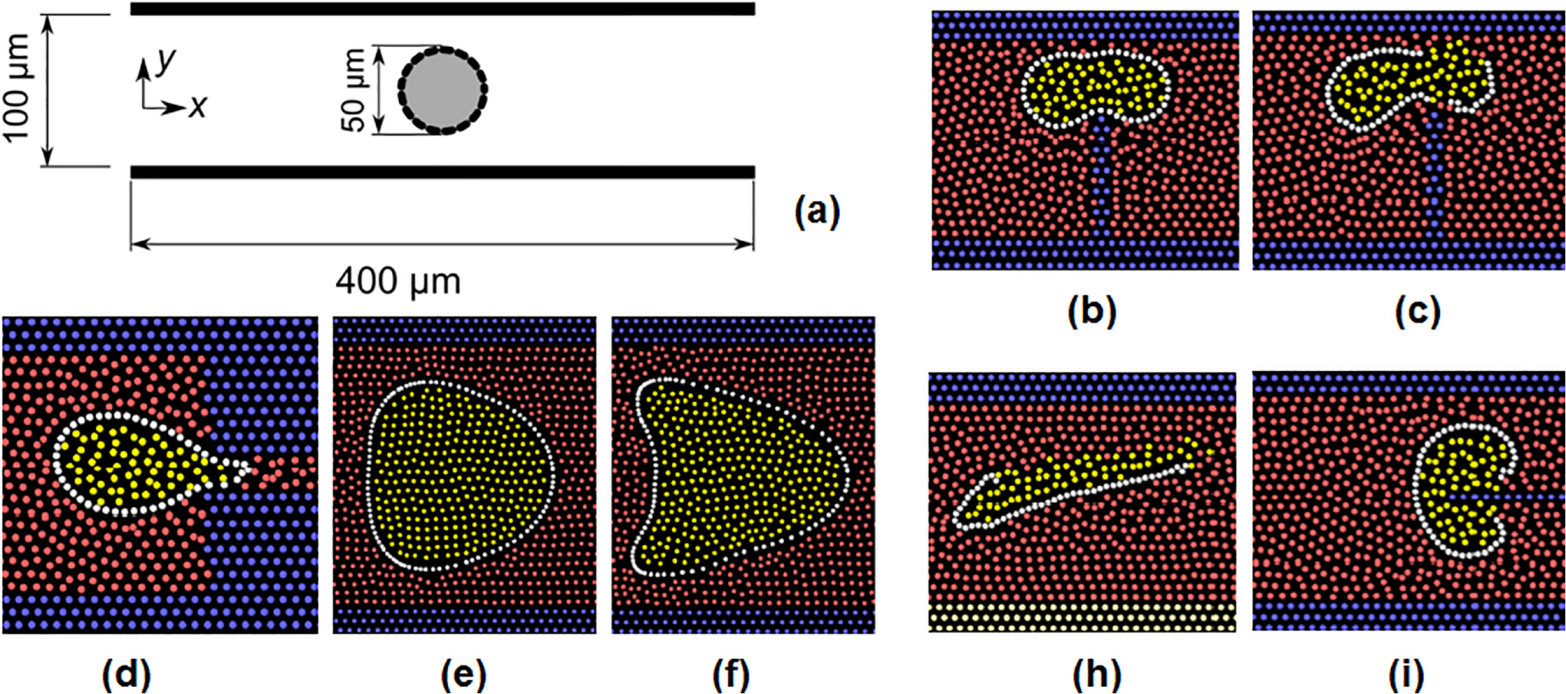
Cells, vesicles and capsules under various flow conditions.

This case has been extensively investigated in [15] and it is here only mentioned for completeness. The geometry is shown in Fig 6A. Boundary conditions in the *x* direction are periodic. This means that when a particle exits the channel from one end, it re-enters from the opposite end. The liquid is divided in 2048 fluid particles with a mass *m* = 2.5 10^–8^ kg initially located at a distance ΔL = 5 10^–6^ m. The cell is constituted by a membrane discretized with 48 elemental particles and the internal cytoplasm (61 elemental particles). The density of the liquid is *ρ* = 1000 kg m^-3^, the viscosity *μ* = 0.1 kg m^-1^ s^-1^, the smoothing length *h* = 1.18 10^–5^ m, *r*_0_ = 3.3 10^–6^ m (when breakage is considered, this occurs at *r*_0_ > *r*_max_ = 3.6 10^–6^ m), *k*_*b*_ = 10 J m^-2^; *θ*_0_ = 172.5 rad, *k*_*a*_ = 10^–18^ J, no dihedral potential is considered. The membrane, therefore, has elastic and bending modulus, but since only one layer of particles is used, Poisson’s ratio, shear and torsional modulus are neglected. The L-J parameters used for the solid-liquid interaction (Eq 21) are *σ* = ΔL and ε = 10^–12^ J. The time step adopted is *Δt* = 10^–7^ s and the simulations run for 10^7^ time steps. For simplicity, the properties of the fluid inside the capsule are assumed the same of the external fluid. Since only one cell is simulated no cell-cell interaction is considered; the model, therefore, is a SPH–CGMD hybrid instead of a SPH–CGMD–DEM hybrid.

This case is used to test the model for very flexible particles that easily deform with the flow. Fig 6 gives an overview of the cases investigated. In the case of Fig 6B, 6C and 6I, the flow is driven by an external volumetric, gravity-like force with acceleration *f*_*g*_ = 1 m s^-2^ in the *x* direction. In Fig 6B, an obstacle is placed transversely to the direction of the flow and the (unbreakable) cell must deform in order to pass through the narrowing. Fig 6C shows the same simulation for a breakable cell. Fig 6D considers a restriction on the channel outlet with an external force (*f*_*g*_ = 10 m s^-2^) that pushes the fluid towards the end of the channel. This case replicates a situation typical in biology or medicine when cells or bacteria are captured by an aspiration device for manipulation. Fig 6E and 6F have been used in [23] to study the deformation of cells in confined geometries at various Capillary Numbers and to validate the method by comparing the results with numerical and experimental data in the literature [24]. In Fig 6H the upper wall is put in motion with a velocity *v*_*w*_ = 2∙10^–3^ m s^-1^. The strong shear flow initially deforms the soft particle and, subsequently, tears it off releasing is internal content in the flow. In Fig 6I, finally, a sharp object is added at the end of the channel. The flow pushes the particle towards the sharp object. Initially, the particle deforms, but above a certain pressure the sharp object pierces the external membrane releasing its content.

### Non-spherical particles in Poiseuille flow

**Fig 7:**
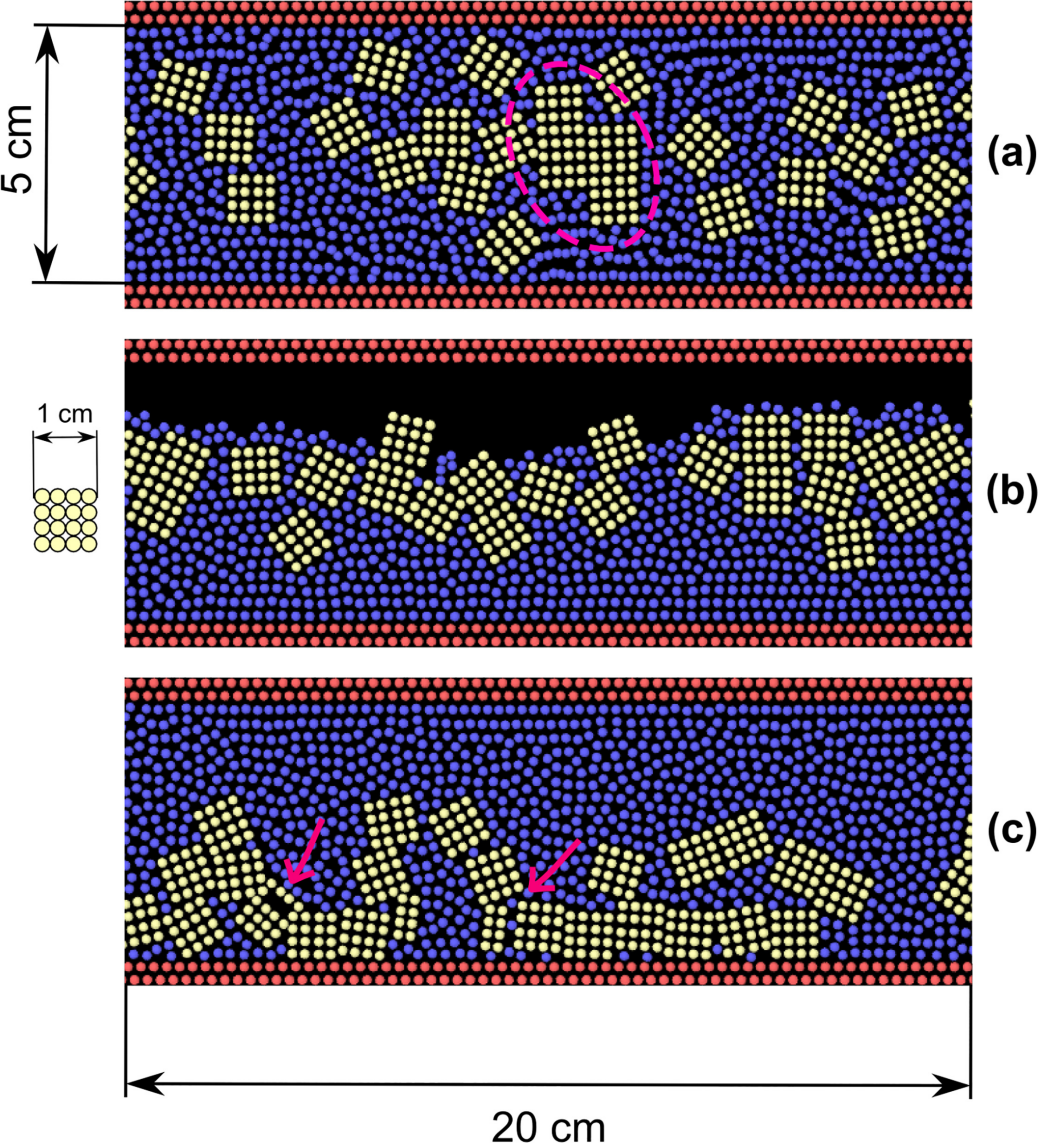
Neutrally buoyant, buoyant and heavy cubic particles in Poiseuille flow.

The second set of simulations considers Poiseuille (*f*_*g*_ = 0.1 m s^-2^) flow with dispersed cubic particles The liquid is water with the same properties as Section 4.1. The geometry is larger (see Fig 7) and the fluid particles have mass *m* = 5.7∙10^–3^ kg. The smoothing length is *h* = 5.6∙10^–3^ m, *r*_0_ = 2.3∙10^–3^ m, *k*_*b*_ = 10 J m^-2^; *θ*_0_ = 90 rad, *k*_*a*_ = 10^–4^ J. This time both normal (Eq 14) and tangential (Eq 17) DEM forces are considered, *k*_*n*_ = 10^5^ J m^-2^, γ_*n*_ = 0.3 s^-1^ *k*_*t*_ = 10^3^ Jm^-2^, γ_*t*_ = 0.2 s^-1^ and μ^s^ = 0.5. The time step adopted is *Δt* = 0.5∙10^–4^ s and the simulations run for 10^7^ time steps. Three cases are investigated. In Fig 7A, we have particles neutrally buoyant. In Fig 7B, the particles have a density of 990 kg m^-3^ and, therefore, they tend to float. In order to highlight one of the features of SPH, namely the facility in handling free surfaces, the pipe is, in this case, only partially filled with water. Surface tension is here not considered, but can be easily introduced in the SPH framework [4]. Fig 7C simulates the case of heavy brittle particles with density 1500 kg m^3^. The solid phase tends to deposit to the bottom of the pipe, but, because the velocity is lower near the walls and higher at the centre, certain particles first are pushed over the other, and then fall on those below. The cubes above hit with all their weight those underneath and, in some cases, break them (breakage is handled as in Section 4.1 with *r*_max_/*r*_0_ = 0.001). This explains the type of fractures observed in Fig 7C.

Despite the fact that simulations are 2D, they still capture some typical features observed in experiments [25] like the formation of temporary clusters of particles that, periodically, are created and destroyed in the flow (highlighted in Fig 7A).

### Solidification and melting

The approach proposed is not limited to moment transfer. In the SPH framework, for instance, we can write the internal energy balance [26] as

$$m_{i}\frac{de_{i}}{dt}=-\sum_{j}\frac{m_{i}m_{j}}{\rho_{i}\rho_{j}}\frac{\left(\kappa_{i}+\kappa_{j}\right)\left(T_{i}-T_{j}\right)}{r_{i,j}^{2}}r_{i,j}\cdot \nabla_{j}W_{i,j}.$$ (22)

**Fig 8:**
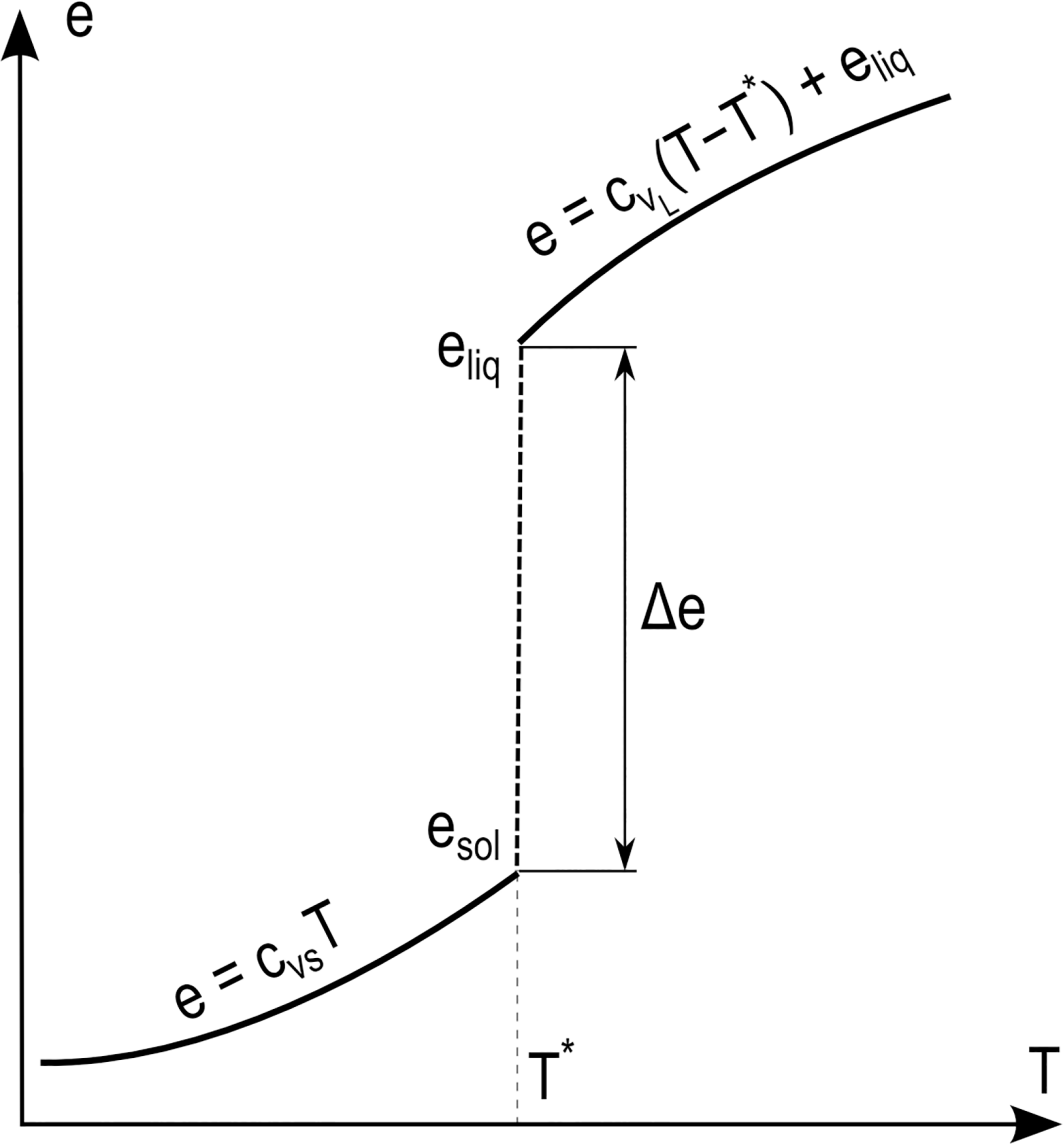
Internal energy versus temperature (assuming *e*_*0*_ = 0 and *T*_*0*_ = 0) in the case of phase transition.

A certain number of new properties such as *e*_*i*_ (internal energy), *T*_*i*_ (temperature) and *κ*_*i*_ (thermal conductivity) are associated to each particle *i* and evolved according to Eq 22. The most general form of Eq 22 would include also the dissipation terms coming from the momentum balance, but these are here neglected. Analogously to Eq (6), an equation of state is required to close Eq (22). Here, we use

$$e_{i}-e_{0}=c_{\text{v}}\;(T_{i}-T_{0}),$$ (23)

where *c*_v_ is the heat capacity, and *e*_0_ and *T*_0_ respectively the reference internal energy and temperature (here both equal to 0). In the case of solidification and melting, however, latent heat (see Fig 8) is involved and this must be considered in the equation of state. Eq (23) therefore must be modified accordingly (considering both *e*_0_ and *T*_0_ equal to 0)

$$\{\begin{array}{c} \begin{array}{l} T_{i}=\frac{e_{i}}{c_{{\text{v}}_{\text{S}}}}\;\text{for}\;e_{i}<e_{sol}\\ T_{i}=T^{*}\;\text{for}\;e_{sol}<e_{i}<e_{liq} \end{array}\\ T_{i}=\frac{e_{i}-e_{liq.}}{c_{{\text{v}}_{\text{L}}}}+T^{*}\;\text{for}\;e_{i}>e_{liq} \end{array}$$ (24)

where c_vs_ is the heat capacity of the solid, c_vL_ the heat capacity of liquid, *T* ^*^ the transition temperature, *e*_*liq*_ the internal energy of the liquid at *T* ^*^ and *e*_*sol*_ the internal energy of the liquid at *T* ^*^.

**Fig 9:**
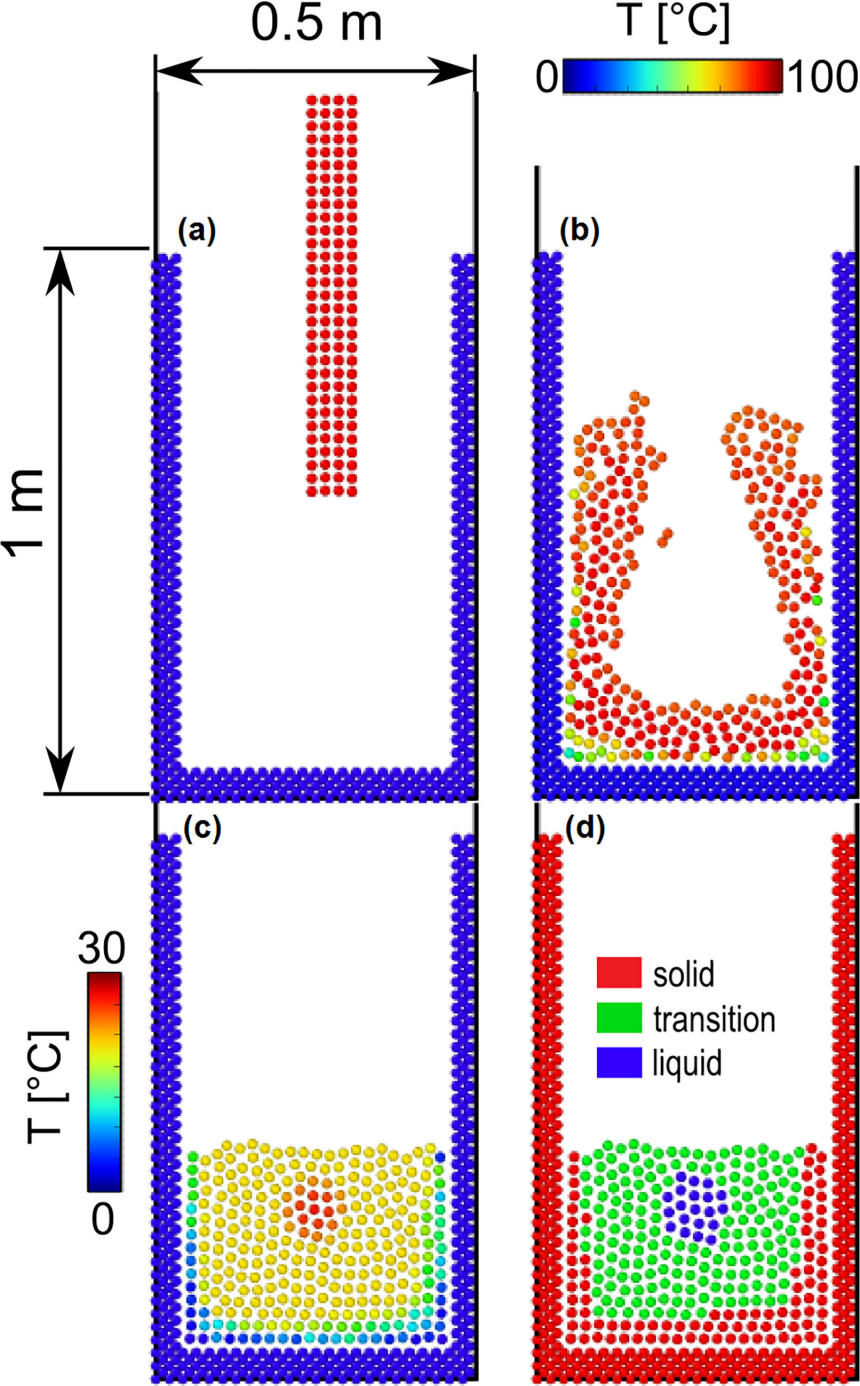
Casting with slow solidification. Particles coloured according to their temperature in (a), (b) and (c), according to their state in (c).

In Fig 9, we consider a liquid with *ρ* = 1000 kg m^-3^ and thermal conductivity *κ* = 1 J s^-1^ m^-1^ K^-1^ that solidifies at *T* = 25°C. The mass of each liquid particle is *m* = 6.2∙10^–1^ kg, its smoothing length is *h* = 4∙10^–2^ m, *e*_*sol*_ = 50 J kg^-1^, *e*_*liq*_ = 100 J kg^-1^, c_vs_ = 2 J kg^-1^ K^-1^ and c_vL_ = 1 J kg^-1^ K^-1^. The time step adopted is *Δt* = 10^–3^ s and the simulations run for 10^5^ time steps. Initially the liquid is at *T* = 100°C, while the walls are at *T* = 0°C (*κ*_*WALLS*_ = 100 J s^-1^ m^-1^ K^-1^). The liquid is poured into the mould and exchange heat with the walls as illustrated in Fig 9. Solidification is handled in the following way: when an elemental particle reaches an internal energy lower than *e*_*sol*_ = 50 J kg^-1^, it is labelled as ‘solid’; at this point, the algorithm searches for neighbour solid particles and, if it finds any, it creates a new bond between them. Two particles are considered neighbour if they are located within a cut-off distance ΔL = 2.5 10^–2^ m. The bonds are here assumed harmonic (Eq 11) with *r*_0_ = 2.5 10^–2^ m and *k*_*b*_ = 10^5^ J m^-2^. Once the particle solidifies, it cannot actively create new bonds, but it can be included in bonds formed by other solidifying particles. If the temperature of a solid particle rises above *e*_*liq*_ = 100 J kg^-1^, it melts again, it is labelled as ‘liquid’, and all its bonds are destroyed. During solidification, the particle is considered a SPH particle until complete solidification (*e* < *e*_*sol*_) occurs and a DEM particle afterwards. During melting, the particle is considered a DEM particle until complete melting (*e* > *e*_*liq*_) occurs and a SPH particle afterwards. A gradual change of the rheological properties of the material during solidification/melting is also possible as done later on for lava flows.

Heat exchange with air is also considered. We cannot use Eq (22) for this because air particles are not directly included in the simulations. Particles located at the surface of the liquid, therefore, exchange heat with a virtual isothermal medium according to Newton’s law of cooling

$$\frac{dT}{dt}=-k_{T}(T-T_{air}).$$ (25)

with *k*_*T*_ = 0.02 s^-1^ and *T*_*air*_ = 0°C. A particle is located at the liquid surface if its coordination number, calculated using *h* as cut-off, is lower than 5.5. Fig 9A, 9B and 9C show the fluid entering the mould and then gradually solidifying. Fig 9D shows the same situation of Fig 9C, but it highlights the formation of three zones. The blue zone indicates the liquid at *e* > *e*_*liq*_ and *T* > T*, the green zone indicates the transition at *e*_*liq*_ < *e* < *e*_*liq*_ and *T* = T* and the red zone indicates the solid at *e* < *e*_*liq*_ and *T* < T*.

**Fig 10:**
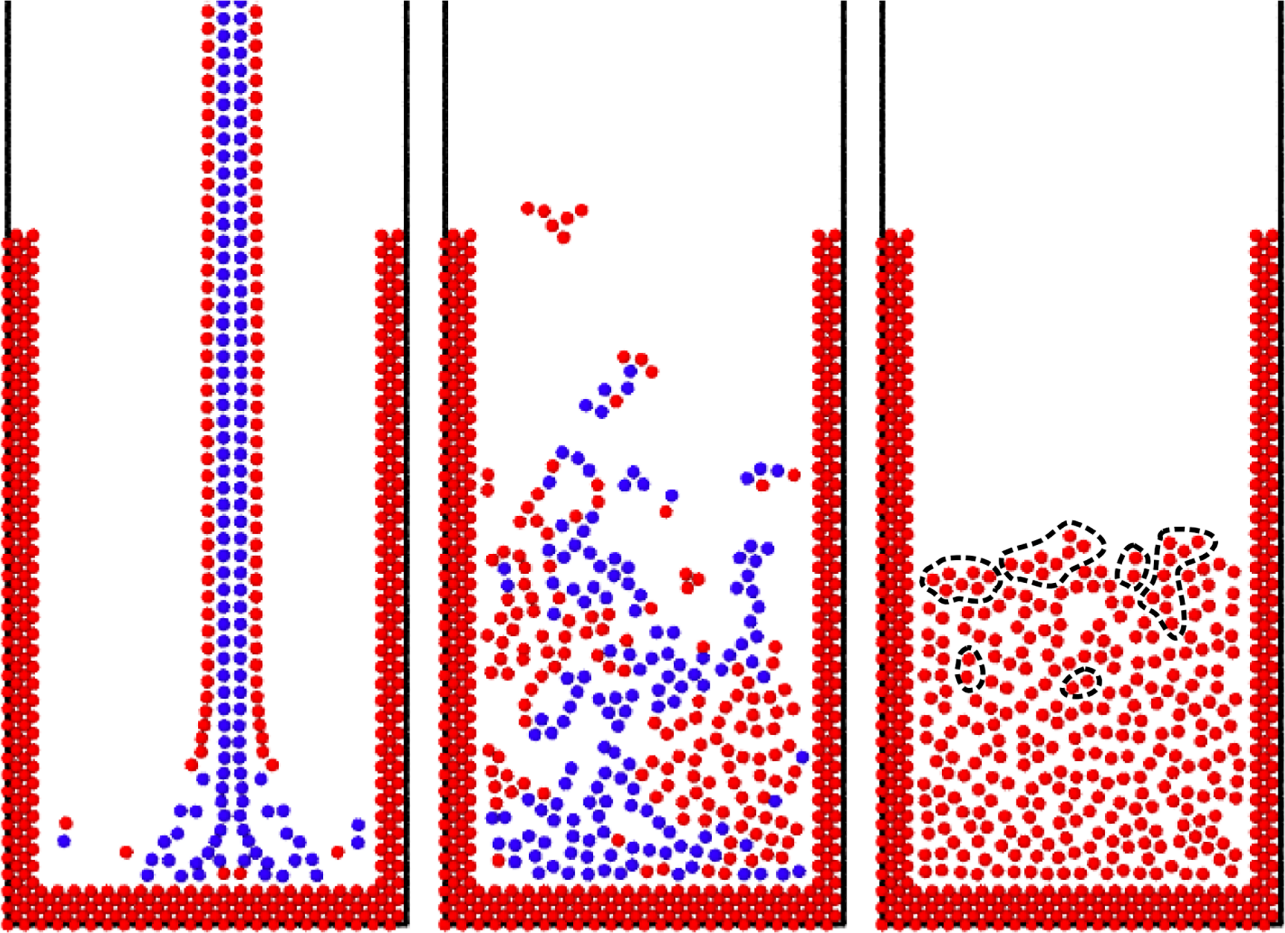
Casting with fast Solidification (particles coloured according to their state: red = solid, blue = liquid).

In Fig 9, the heat exchange with the surface is lower than that with the walls. In a second simulation (Fig 10), *k*_*T*_ is increased to 0.5 s^-1^. This creates an exceptionally high heat loss from the liquid surface. This is certainly a very extreme situation, but, at the same time, a good test for checking the reliability of our model. In this case (Fig 10), the liquid starts to solidify and a solid crust forms on the casting even before it touches the mould. In order to enhance this effect, the latent heat has been neglected (*e*_*sol*_ = *e*_*liq*_). When it reaches the bottom, we observe two phenomena. The first is the mechanical breaking of the particles constituting the crust (breakage is handled as in Section 4.1 with *r*_max_ = 4∙10^–2^ m). The second is the partial re-melting of the crust once it is put into contact with the hot bulk flow. Both solid fragments and liquid drops are then projected into the air. The drops rapidly cool due to heat transfer with air. Colliding solid particles interact via DEM forces with *k*_*n*_ = 10^4^ J m^-2^, γ_*n*_ = 0.3 s^-1^ *k*_*t*_ = 10^3^ J m^-2^, γ_*t*_ = 0.2 s^-1^ and μ^s^ = 0.5. When all the liquid is solidified (see last Fig 10), the resulting solid is very porous and it is constituted of fragments (highlighted in Fig 10), which are small agglomerates with no links with the rest of the structure. The majority of these are at the surface because they landed on the block after it has already solidified, but some of them can also be found embedded in the bulk. The rest of the structure is a unique block, but formed from half-solidified fragments and, for this reason, it has a very porous structure. As mentioned, the case of ultrafast solidification is extreme and it has been introduced mainly for testing purposes. Despite this, the model captures the correct phenomenology of real applications where such a rapid cooling actually occurs [27].

### Cleaning processes

In this example, the removal of protein-based soils deposits on hard surfaces is simulated. This is a typical cleaning problem and its phenomenology is known in detail [28]. Initially the protein swells due to water absorption. Then, when the moisture content reaches a certain level, the soil structure is weakened and the removal of the substance occurs. In order to simulate this process, a new property, water concentration *c*_*i*_, is introduced in the SPH part of the model. Each elemental particle, therefore, besides its position and velocity, also carries an elemental concentration. Analogously to Eq 22, the diffusive mass balance of water can be written in the following way

$$\frac{dw_{i}}{dt}=-\sum_{j}\frac{m_{i}m_{j}}{\rho_{i}\rho_{j}}\frac{\left(D_{i}+D_{j}\right)\left(c_{i}-c_{j}\right)}{r_{i,j}^{2}}r_{i,j}\cdot \nabla_{j}W_{i,j},$$ (26)

where *w*_*i*_ is the mass of water in the particle and *D*_*i*_ (diffusion coefficient) are associated to each particle *i* and evolved according to Eq 26. As happened with Eq (6) and Eq (22), also for Eq (26) we need a closure term, which, in this case, is simply

$$w_{i}=c_{i}\frac{m_{i}}{\rho_{i}}.$$ (27)

**Fig 11:**
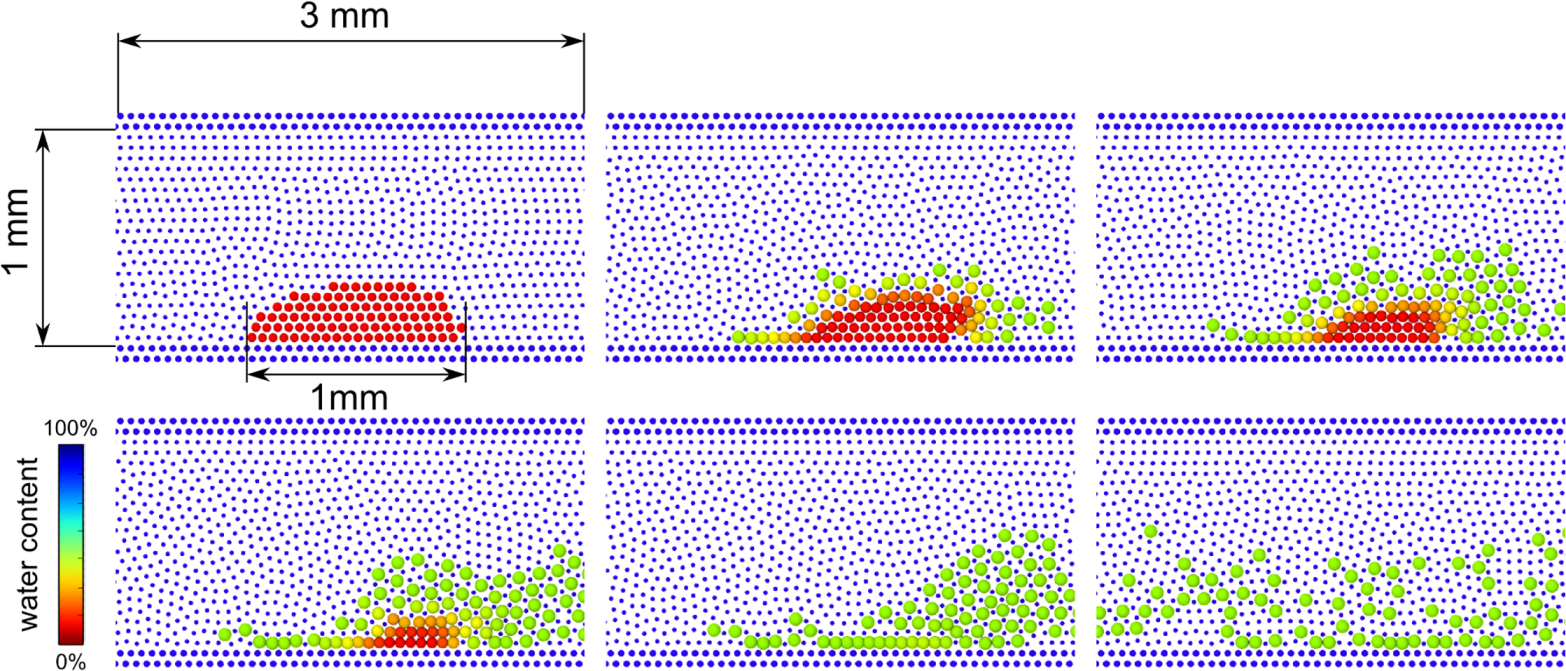
Swelling and erosion of a protein-based soil deposit (particles coloured according to their water content).

In theory, the water particles that exchange mass with the protein should reduce their mass. This phenomenon, however, has been neglected here because of the excess of water in the system. In Fig 11, we consider a liquid with *ρ* = 1000 kg m^3^, the mass of each liquid particle is *m* = 2.3∙10^–6^ kg and its smoothing length *h* = 1.1∙10^–4^ m. A velocity gradient is imposed to the water by moving the upper wall with *v*_*W*_ = 2∙10^-4^m s^-1^. Van der Walls adhesion forces between soil particles are modelled with a Lennard-Jones potential with *σ* = 5∙10^–5^ m and ε = 7∙10^–11^ J. Adhesion between soil particles and the surface is modelled in the same way but with ε = 5∙10^–10^ J. The surface-soil interaction force, therefore, is stronger than the soil-soil one. The time step adopted is *Δt* = 10^–6^ s and the simulations run for 5∙10^7^ time steps. The soil particles initially have density *ρ* = 1800 kg m^3^ and diameter of 4.7∙10^–5^ m. During the simulation they adsorb water according to Eq (26) (*D* = 10^–9^ m s^-2^), swell and their volume increases proportionally to the water absorbed. Due to the presence of water, the adhesion forces among soil particles decrease linearly with the concentration of water. When the concentration reaches 0.5, there is no adhesion left and the particles are washed away by the flow. The results shown in Fig 11 are perfectly consistent with the known physics of the problem [28].

### Lava flow on inclined plane

**Fig 12:**
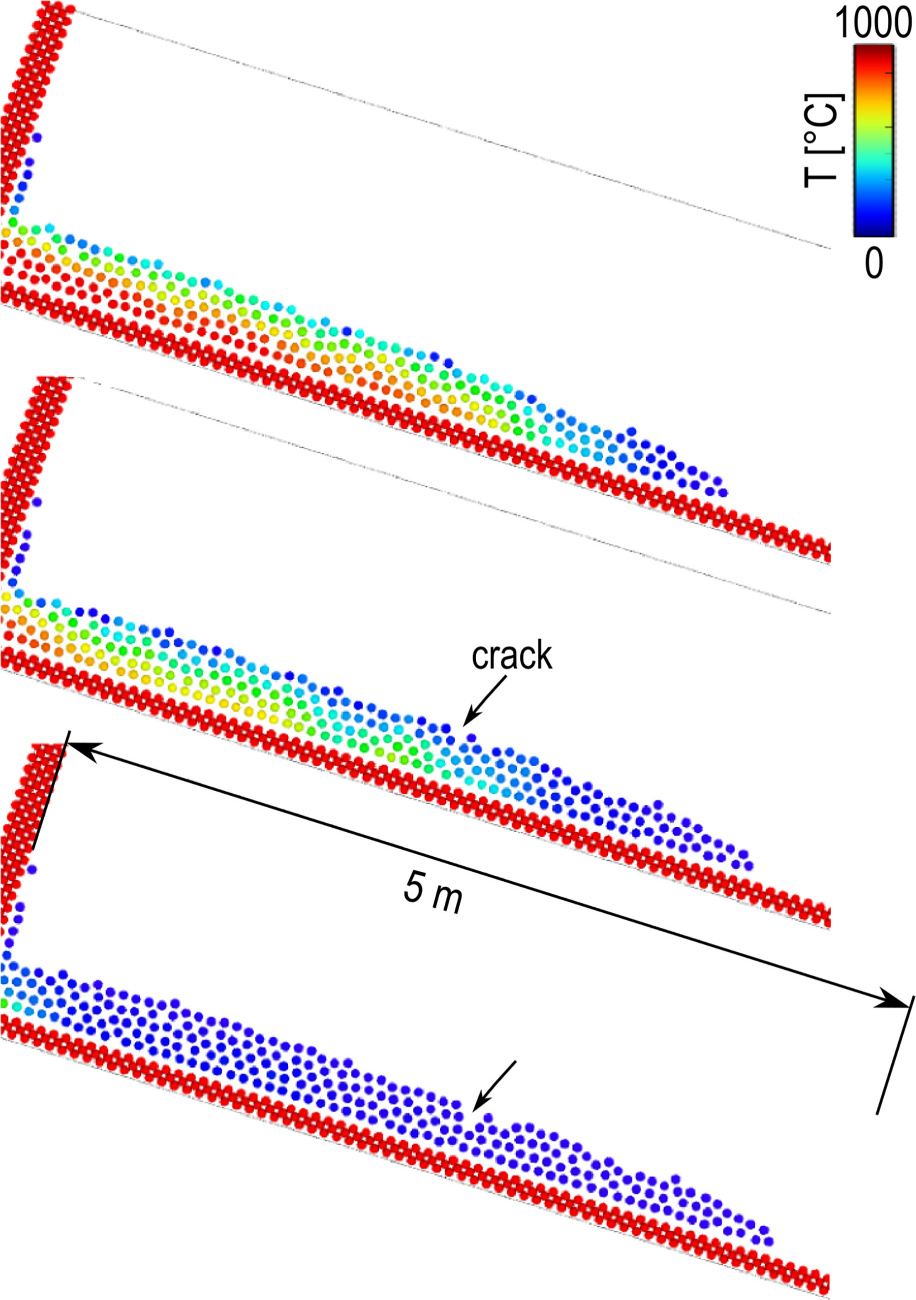
Lava flow on an inclined plane (particles coloured according to their temperature).

In this section, we consider a liquid with *ρ* = 2000 kg m^3^, heat capacity c_v_ = 1 J kg^-1^ K^-1^, viscosity 1.0 kg m^-1^ s^-1^ and thermal conductivity *κ* = 1 J s^-1^ m^-1^ K^-^ that flows on an 30° inclined plane. The mass of each liquid particle is *m* = 1.25 kg and smoothing length *h* = 4∙10^-2^m. Initially the liquid is at *T* = 1000°C and solidification occurs at *T* = 500°C. The walls are excluded by the mass transfer therefore cooling happens only because of Eq (25) where *k*_*T*_ = 0.02 s^-1^ and *T*_*air*_ = 0°C. The time step adopted is *Δt* = 10^–4^ s and the simulations run for 10^7^ time steps. Solidification re-melting and solid breakage are handled similarly to Section 4.4. Once the particle are solid they interact via DEM forces with *k*_*n*_ = 10^6^ J m^-2^, γ_*n*_ = 0.3 s^-1^ *k*_*t*_ = 10^4^ Jm^-2^ and γ_*t*_ = 0.1 s^-1^. Latent heat is not considered in this case, because often lava solidification involves glass transition [29], which does not produce latent heat. In theory, the heat capacity coefficient should be function of the temperature, but this has been here neglected. Fig 12 shows three snapshots of the simulation. Cooling and solidification begin at the liquid surface and an external solid crust forms. The bulk, however, is still fluid and its motion generates tensions in the crust. Because of this, the crust breaks and cracks appear. This behaviour is consistent with observation of lava flow [30].

**Fig 13:**
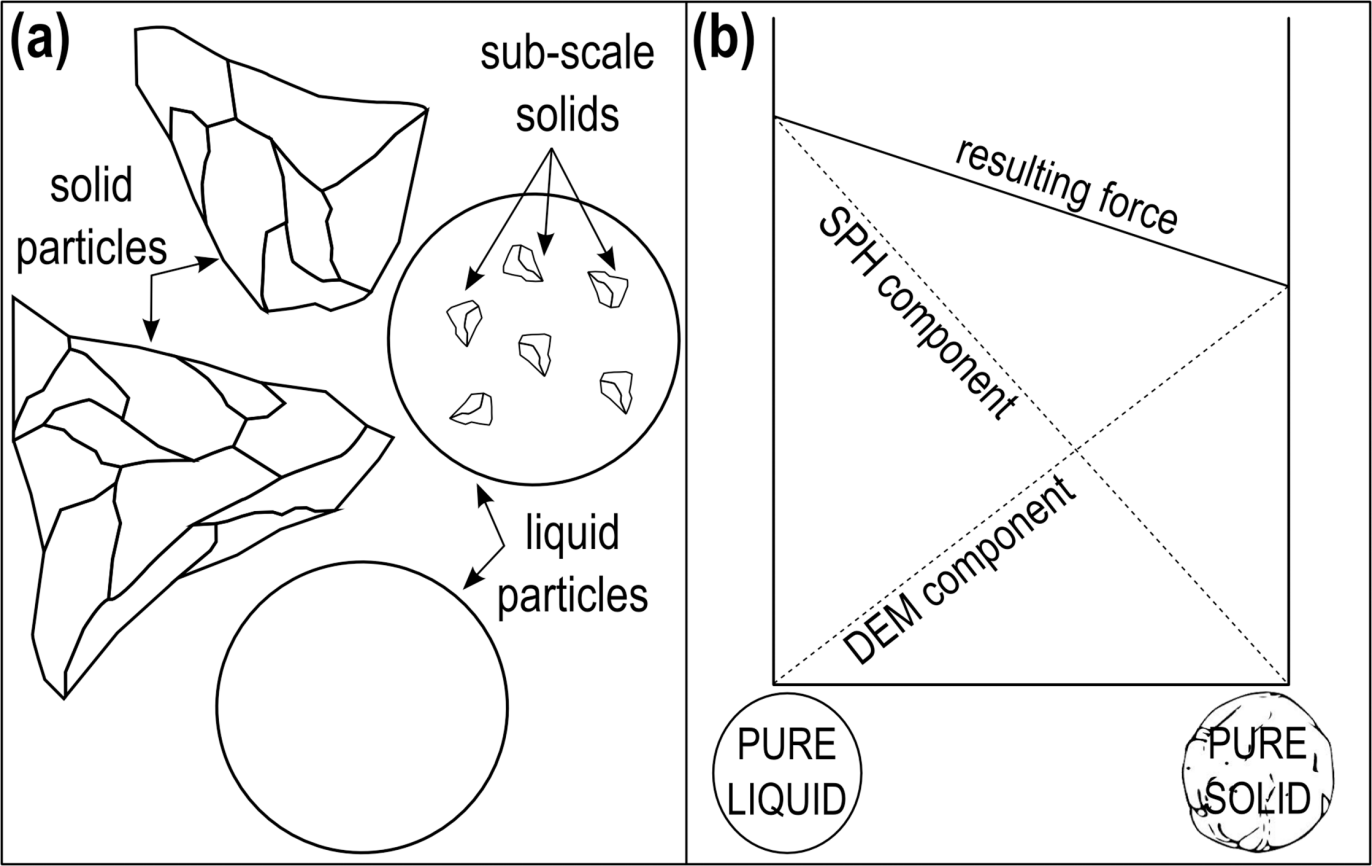
Mixing SPH and DEM forces to account for sub-scale solids.

We use this example also for illustrating a possible way to approach complex rheology in our framework. Let’s suppose that the liquid *per se* is Newtonian, but it contains a large quantity of solid particles that confer to the flow a Bingham-like behaviour. These solid particles are created during solidification: some of them become large enough to be represented by computational particles, others never reach this minimal size (see Fig 13A). We call these small particles “sub-scale solids”. The smallest portion of matter that the model can handle as an independent solid is of the size of elemental particles. Smaller sub-scale solids, however, can contribute considerably to the rheology of the flow. In order to embed the effect of these sub-scale solids in the liquid, we employ hybrid particles with an intermediate behaviour between SPH and DEM (see Fig 13B). At 1000°C, the particle is completely liquid and we only consider SPH forces. At 500°C, the particle is completely solidified and we only consider DEM forces. In between, we assume an intermediate behaviour where SPH and DEM forces are mixed together.

This method can prove very useful for the simulation of complex fluids where the complexity of the rheology depends on dispersed particles with a wide size distribution: the larger particles are handled directly as real solids, while sub scale solids with the mixing procedure described above.

### Particle separation

**Fig 14:**
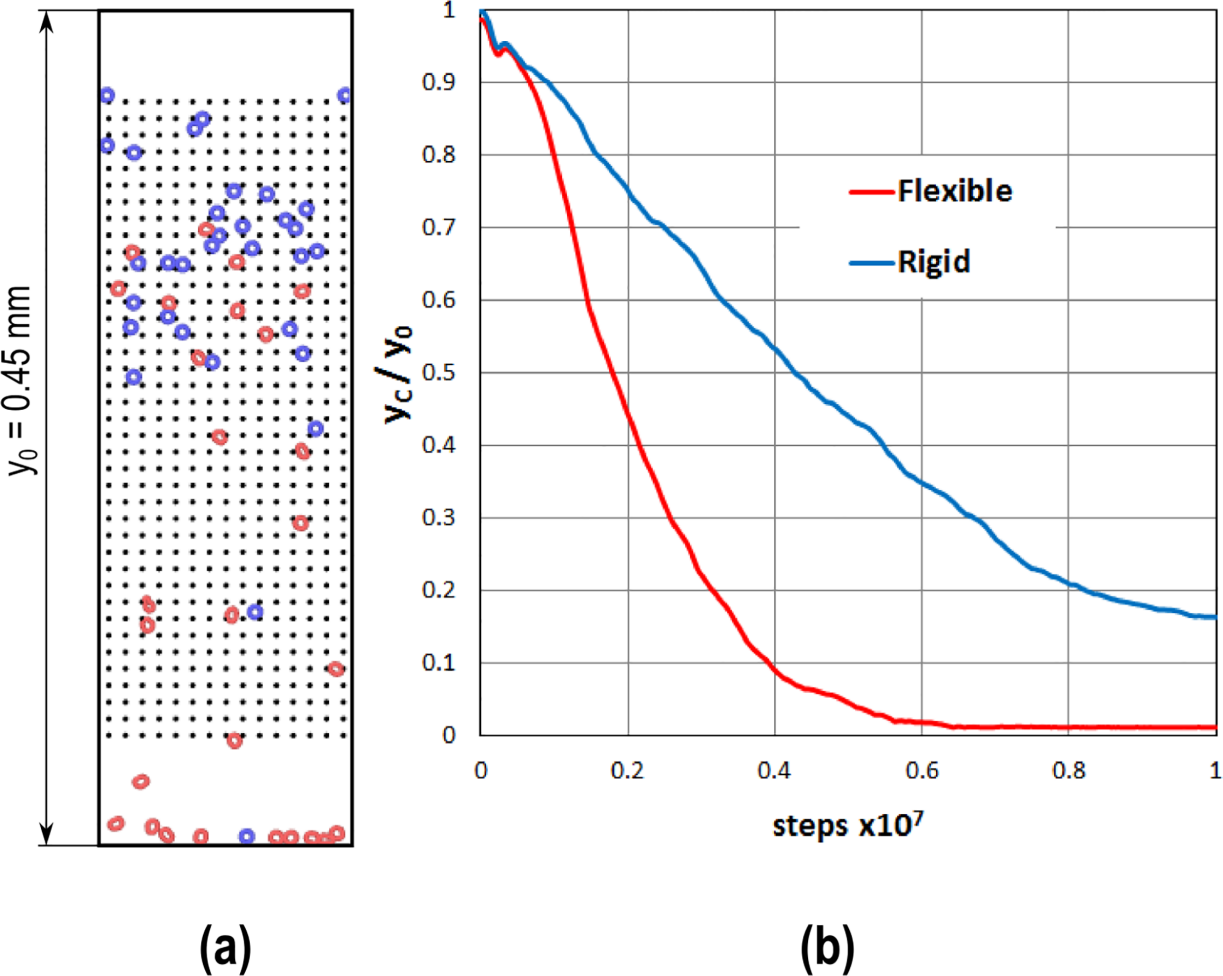
Plinko-chip for cell separation (a); evolution of the center of mass for flexible and rigid cells (b).

There are different techniques for particle separation based on particles’ differences in size, weight or shape. In this section, we use the DMHS to simulate a “Plinko-chip” (Fig 14A) that can separate particles, whose only difference consists in their rigidity. We consider here two types of particles: one is more flexible than the other, but, except for this, all the remaining properties are exactly the same. This application refers to a possible cell separator that can sort cancer cells from healthy cells, which only differs for their rheological properties. We consider cells with radius of 10 μm moving in aqueous environment. The length of the chip is y_0_ = 1.5 mm, the width x_0_ = 0.45 mm and the distance between two adjacent pins 30 μm. The cells start at the top of the Plinko-chip and move towards the bottom due to a gravity-like force. The external water is stagnant and, therefore, no SPH component is considered in this model, which, therefore, is a DEM–CGMD hybrid instead of a SPH–CGMD–DEM hybrid. Water resistance is considered as Stokes’ drag acting on each particle

$$F_{i}^{drag}=-6\pi \mu r_{i},$$ (28)

where *r*_*i*_ is the radius of particle *i*. Only the external membrane of the cell is considered and assembled with 16 computational particles with density *ρ* = 1000 kg m^-3^. Adjacent particles belonging to the same cell are bonded together with a harmonic bond with *r*_0_ = 3.9∙10^–6^ m and *k*_*b*_ = 10^–2^ J m^-2^, and an angle bond with *θ*_0_ = 157.5 rad and *k*_*a*_ = 10^–15^ J for the rigid cells and *k*_*a*_ = 10^–16^ J for the flexible cells. The value of *k*_*a*_ is the only difference between the two types of cell and confers a different flexibility to each group. DEM forces between a cell and (i) other cells, (ii) the pins of the Plinko-chip and (iii) the walls of the chip are considered with *k*_*n*_ = 10 J m^-2^, γ_*n*_ = 0.3 s^-1^ *k*_*t*_ = 10 J m^-2^, γ_*t*_ = 0.3 s^-1^ and μ^s^ = 0.5. The time step employed is Δ*t* = 10^–7^ s. Fig 14 shows an example of simulation: the red cells are the flexible ones, the blue the rigid. Because of the small size of the cells and the presence of water resistance, the body force required to move the cells in the chip must be higher than gravity (here *f* = 100 m s^-2^). In practice, this can be achieved by introducing in the cells specifically designed magnetic nanoparticles [31] and employing magnetic forces. While they move along the channel, cells are separated because the flexible ones can more easily pass in the narrow gaps through the pins. Fig 14B shows the centre of mass y_C_ of the flexible and rigid cells at each time step. At the beginning all the cells are randomly allocated in the upper region and y_C_ is approximately the same for both types. The flexible cells, however, descend faster along the channel and, therefore, their centre of mass decreases faster than that of the rigid cells. At the end of the simulation, when all the cells are collected at the bottom of the chip, they mix again, but there is a period of time, where a certain degree of separation is achieved as indicated in Fig 14B.

## Conclusions

In this article, a methodology based on CGMD, SPH and DEM is presented and discussed. This approach links together multiple discrete models in a hybrid fashion and, for this reason, has been named the discrete multi-hybrid system. The main goal of this paper is to show how this approach can be used to simulate a large variety of solid-liquid dispersed flows. Here we have focused on specific applications involving deformable, breakable, melting/solidifying and swelling particles, but other examples such as filtration or erosion could have been easily added. The model has been tested in various different situations and sometimes under very extreme conditions and, in all cases, the simulations correctly reproduced the expected physics of the system. It is also important to highlight the variety of scales, from microns to meters, covered by the examples.

In the case of casting and cleaning, moreover, the use of SPH for mass and heat transfer implies a multi-scale approach, where the motion of solidified drops and soil particles is accomplished particle by particle, while heat and mass transfer are handled almost in a continuum fashion. This approach is conceptually similar to certain atomistic-continuum methodologies [32] with the difference that here scale separation is achieved within a discrete framework and by adopting different types of **F**_*i*,*j*_ forces rather than by physically separating the discrete and the continuum domains. This, of course, poses a limit at the scale separation manageable by the model especially in terms of time-scale. As a consequence, we don’t expect this technique, to cover phenomena characterized by extremely large scale separations as in continuum-atomistic methods.

The lava flow example introduces yet another multi-scale solution. In order to take into account the progressive formation of sub-scale solids in the solidifying liquid, the properties of the elemental particles have been gradually transformed from SPH to DEM. This approach can be employed to simulate complex fluids where the rheological properties depend on dispersed solids of different size. The larger solid grains can be simulated directly, while the effect of the smaller sub-scale solids can be included collectively by introducing a DEM component in the SPH-particles.

The examples considered in the paper have been limited to scales where Brownian motion is not directly observable. This restriction, however, can be removed and the effect of fluctuating hydrodynamics can be easily included in the model by, for instance, substituting SPH with Smoothed Dissipative Particle Dynamics. Finally, the possibility to employ computational particles with variable sizes, as feasible in the SPH method [4], has the potential of further enhance the multi-scale capabilities of our multi-hybrid system.
